# Impact of interventions on the quality of life of cancer patients: a systematic review and meta-analysis of longitudinal research

**DOI:** 10.1186/s12955-023-02189-9

**Published:** 2023-10-11

**Authors:** Long Bao Nguyen, Linh Gia Vu, Thanh Thien Le, Xuan Thanh Nguyen, Nam Gia Dao, Duy Cao Nguyen, Trang Huyen Thi Dang, Thuc Minh Thi Vu, Laurent Boyer, Guillaume Fond, Pascal Auquier, Carl A. Latkin, Melvyn W.B. Zhang, Roger C.M. Ho, Cyrus S.H. Ho

**Affiliations:** 1 Institute for Preventive Medicine and Public Health, Hanoi Medical University, Hanoi, 100000 Vietnam; 2 Institute for Global Health Innovations, Duy Tan University, 550000 Da Nang, Vietnam; 3 Faculty of Medicine, Duy Tan University, Da Nang, 550000 Vietnam; 4 Department of Biomedical Engineering, Tufts University, 200 College Avenue, Medford, MA 02155 USA; 5 Hue Central General Hospital, Hue, 52000 Vietnam; 6 Institute of Health Economics and Technology (iHEAT), Hanoi, Vietnam; 7 Research Centre on Health Services and Quality of Life, Aix Marseille University, 27, boulevard Jean-Moulin, CEReSS, Marseille cedex 05, 3279, 13385 France; 8 Bloomberg School of Public Health, Johns Hopkins University, Baltimore, MD 21205 USA; 9 Lee Kong Chian School of Medicine, Nanyang Technological University Singapore, Singapore, Singapore; 10 Department of Psychological Medicine, Yong Loo Lin School of Medicine, National University of Singapore, Singapore, 119228 Singapore; 11 Institute for Health Innovation and Technology (iHealthtech), National University of Singapore, Singapore, 119077 Singapore

**Keywords:** Quality of life, HRQL, Cancer, Interventions

## Abstract

The impact of cancer interventions has been conducted in several research due to the significant burden of this non-communicable disease. The interventions that played an important role in the improvement of the patient’s quality of life (QoL) and health-related quality of life (HRQL) can be classified into two main groups: pharmaceutical and non-pharmacological methods. However, studies so far often analyze a specific group of interventions for specific types of cancer. Thus, in this systematic review and meta-analysis, we synthesized the overall impact of cancer interventions on patients’ quality of life in several cancers.

In this research, we followed the Preferred Reporting Items for Systematic Review and Meta-Analysis Protocols (PRISMA-P) to search the longitudinal original research on the Web of Science (WOS) database. After that, the Newcastle-Ottawa Scale (NOS) and Jadad Scale were used to assess the quality of non-randomized control trials and randomized control trials, respectively. Then, the characteristics of the included studies were described in the six main fields table and the random effect model with robust estimation was applied to analyze the impact of interventions on the health utility of patients.

From the database, 122 longitudinal original research were included in the meta-regression, with most of them having high or fair quality. The European Organization for the research and treatment of cancer scale for quality of life (EORTC-QLQ) was the most used health utility measurement at 65.15%. In the adjusted effect models, the Visual Analogue Scale (VAS) had significant statistics in all models when we compared it with the EQ-5D Scale (*p* < 0.05) and several types of cancer such as breast, lung, and prostate cancer had significant statistics when comparing with hematological cancer in the model types of cancer (*p* < 0.01). Moreover, radiotherapy, screening, and a combination of chemotherapy and best supportive care also had significant statistics (*p* < 0.01) in the model of interventions when compared with radiotherapy applied only. Our research can suggest a vital combination of both pharmaceutical and non-pharmacological interventions to improve the quality of life of some common types of cancer patients.

**Supplementary Information:**

The online version contains supplementary material available at 10.1186/s12955-023-02189-9.

## Introduction

Cancer remains one of the biggest health risks: the number of new cancer cases and cancer-related mortalities is continuously growing in several countries. Data from the World Health Organization (WHO) showed that in 2020, there were nearly twenty million people newly diagnosed with cancer and more than ten million people died due to cancer [1]. Breast cancer is the most common type of cancer with more than two million new cases, followed by lung cancer, colon rectum cancer, gastric cancer, prostate cancer, and skin cancer, each of them having more than one million new cases [1]. Furthermore, this figure was estimated to rise sharply as one of the most severe modern non-communicable diseases in the next decades. Cancer mortality is also high in developing countries. For example, the Vietnamese Ministry of Health reported that there were approximately two hundred thousand new cancer patients in 2020 and that more than half of them died with a death rate of nearly sixty per cent [2]. Moreover, the World Health Organization also reported that there were nearly six million people in developing countries who can die because of this disease at nearly 70 per cent [1, 3]. Vietnam, Lao, Cambodia, Myanmar, Thailand, and some other South-East Asia have some similar characteristics in cancer epidemiology. Specifically, the percentages of lung and liver cancer were highest in men, while cervical and breast cancer were equally common in women with more than two million new cancer cases in 2022 [4, 5]. Therefore, Vietnam and some other countries may have more effort to reduce the burden of cancer in the future [6].

Besides the high mortality rate, cancer can also significantly affect the quality of life (QoL) and the health-related quality of life of cancer patients and their families. Specifically, the severe and long-term impact of cancer means that not only the mental and physical health of the patients but also their financial status can deteriorate. The financial burden may be a catastrophe for patients in both developing and developed countries. The total cost of cancer treatment is estimated at more than two hundred billion dollars in 2009 and is expected to double in 2040 [7]. Therefore, allocating resources to improve the effectiveness of cancer interventions is extremely necessary to decrease the burden of cancer in the next few decades.

However, there have been some remarkable developments in cancer interventions recently thanks to the application of modern technology and preventive medicine. For instance, the effectiveness of immune checkpoint therapy, personalized medicine, and new gene sequencing can be the more effective therapy. Cancer interventions may be largely grouped into two categories: pharmaceutical and non-pharmacological approaches. The pharmaceutical interventions may include treatments that use direct drug, biological, or chemical intervention, or clinical interventions such as surgery [8]. On the other hand, non-pharmacological interventions can be considered as indirect methods to raise morale, alleviate mental health problems, improve the mental life of the patient, and sometimes include the caregivers [13]. Screening and preventive programs are the first vital interventions that need to combine pharmaceutical and non-pharmacological methods to eliminate cancer. These methods are the important first steps that should be widely adopted, especially among the high-risk groups such as citizens with a history family of cancer, radio, and chemo working condition, and the elderly [9, 10]. Regarding the pharmaceutical group, chemotherapy, radiotherapy, and surgery are the most common types of interventions to limit tumor growth and metastasis. While these methods are mainly responsible for the increase in life expectancy of cancer patients, they also have significant side effects that could decrease the quality of life of patients [11]. Other critical pharmaceutical interventions such as immunotherapy, gene therapy, targeted drugs, bone marrow transplant, and hormone therapy can have varying degrees of effectiveness on cancer treatment, despite the dramatically higher cost [12]. Although the patient’s total number of added life years is expected to increase, the estimated quality of life per year may decrease due to the side effects of interventions. Therefore, It may be necessary to assess the quality of life outcome of cancer patients when they received the interventions [8, 12].

Regarding non-pharmacological interventions, palliative care is recommended to be combined with other curative interventions. Cancer often has a dramatically negative impact on the psyche of the patients and their families. Palliative care, which can be applied from the early stage of cancer detection to end-of-life care, is useful to decrease the psycho burden of cancer patients [13]. Additionally, palliative care also focuses on coping with the cancer treatment process such as decreasing the side effects and relieving pain. Therefore, the application of palliative care measures can contribute to improving patients’ treatment outcomes. Indeed, the combination of pharmaceutical and non-pharmacological interventions can be more effective in treating cancer [14]. For example, a meta-analysis conducted in 2020 emphasized the necessity of combining exercise and nutrition interventions during curative treatment [15]. They found that there were four of six included studies designed to assess the effect of the combination of nutrition and exercise interventions [15]. Thus, to ensure the best quality of life and the health-related quality of life for cancer patients and family members, a regime consisting of curative and palliative care was recommended.

The impact of interventions on the quality of life of cancer patients has been discussed in several research. However, studies conducted so far are often limited in scope: only focusing on specific one or two groups of interventions on a small group of cancer. For instance, the previous research in 2017 only focused on Asian patients with breast cancer under different treatment methods [16]. In this study, they gave information about the poorer health-related quality of life of Asia cancer patients compared with the general population [16]. At the same time, another article in 2017 mentioned the role of chemotherapy for advanced gastric cancer [17]. Although they found that the combination of different types of chemotherapy can improve the survival rates of advanced gastric cancer patients, they only focused on a specific type of cancer and intervention [17]. Therefore, we believe that a systematic and comprehensive view of this topic is lacking. The outcomes of cancer interventions are usually presented by the increase of the QoL points when they applied health utility scales such as the European Organization for the research and treatment of cancer scale for quality of life (EORTC-QLQ) or Functional Assessment of Cancer Therapy (FACT) [18, 19]. For instance, the EORTC-QLQ developed in 1987 is one such condition-specific measure quality of life of cancer patients. This scale included three main part: global health status, functional scales, and symptom scales in different version [18]. Besides, the FACT scales was established before 1990 and represented in 5 points Likert scale (0–4 score). This scale can assess the physical well-being, social/family well-being, emotional well-being, and functional well-being of cancer patients [19].

Thus, this systematic review and meta-analysis will be conducted with this objective: To summarize the impact of cancer interventions on the preference-based health-related quality of life of cancer patients.

## Methods

### The process of study selection for systematic review and meta-analysis

**Fig. 1:**
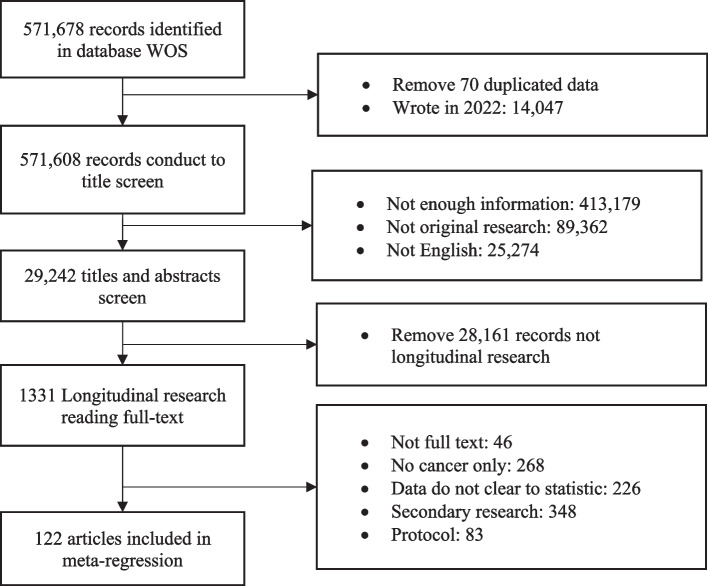
The process of study selection for systematic review and meta-analysis

At first, this systematic review and meta-analysis are based on the description of Preferred Reporting Items for Systematic Review and Meta-Analysis Protocols (PRISMA-P) (Fig. 1) [20].

### Database

The Web of Science (WOS) was chosen as the only database because it has more advantages than other resources of scientific articles such as Pubmed, Scopus, and Google Scholar [21]. To be more specific:

- Publications since the early 21st century were included in this database.
- A high number of full-text paper was included, some of which cannot be found in the other sources.
- Several high scientific impact journal from all over the world was included in this database.

The trends in the literature can be explored easily by some tools in the WOS.

### Eligibility criteria

Data were searched in June of 2022, and the limited publication date was the 31st of December 2021. The reason for this date is the 2022 data cannot show the full general trend and information for this year. Three main keyword terms were applied to search the database Web of Science in Appendix 1, and we used four steps of the searching strategy:

First, the combination of the quality of life and well-being terms was used to search all included studies that mentioned the health utility measurement in topics, abstracts, and keywords. Then, we used the keyword terms for different cancer and interventions to filter one more time. After that, we reviewed all included titles and abstracts of these articles and removed papers with inclusion and exclusion criteria. Finally, the extraction data to conduct the meta-regression of longitudinal studies are in Table 1.

**Table 1:** Data extraction fields

Data fields	Information
General information	Authors
	Countries/ Regions
	Journal
Study characteristic	Study design
	Study duration
Cancer	Type of cancer
Population	Number of populations
Intervention	Type of interventions
	Time of intervention applied
Health utility measurements	Type of Health utility measurements
	Total time of follow up
	Time of each round follow up
	Health utility point (mean, median, SD, SE, 95%CI)

### Inclusion criteria

- The included articles must be published before January 2022 and they were written in English.
- Their content must directly include cancer, intervention, quality of life, and health utility. These studies must be original longitudinal research with full data from patients. They should show the change in the quality of life of cancer patients under the interventions. Non-original research such as systematic reviews, meta-analyses, books, only protocol studies, conference/ poster-only abstracts or E- papers, letters, opinion pieces, editorials, and conference proceedings were excluded. Moreover, ecological studies and cross-sectional studies were not included in this meta-analysis.
- The health utility points of patients in the included research should be reported from the beginning of receiving cancer interventions to the end of follow-up.
- On the results of included studies, they should report the data clearly in the table with mean, median, standard deviation, standard error, 95% confidence interval, and interquartile range at different times of follow-up. The other types of data description such as only figures and missing statistical information on health utility points were excluded.

### Data analysis

The titles, abstracts, and full text were read carefully by two reviewers and checked with the inclusion and exclusion criteria. The data will be extracted from each included study and added to the Microsoft Excel (Microsoft Corporation 2013) Software.

The Scale of Newcastle-Ottawa was chosen to assess the quality of the non-randomized control trial study and the Jadad Scale is an Oxford scoring system to check the quality of randomized control trial study design [22–24]. As regards the Newcastle-Ottawa Scale, this scale was chosen because it may include some vital aspects to judge a study such as selection, comparability, exposure, and outcome. At this point, each part of this scale has four, two, and three maximum points, respectively. Besides, this scale has been developed for a long time by reputable experts and it was used widely in several studies [22, 23]. The final scores were then divided into three categories:

- Good (3–4 points in the selection section, 1–2 points in the comparability section, and 2–3 points in the outcome/exposure section).
- Fair (2 points in the selection section, 1–2 points in the comparability section, and 2–3 points in the outcome/exposure section).
- Poor (0–1 point in the selection section, 0 points in the comparability section, or 0–1 point in the outcome/exposure section).

Regarding the Jadad Scale, the main reasons for the chosen of this scale are the simplicity, convenience, and accuracy. Furthermore, it can fully assess the important elements of an RCTs study design including randomization, blinding, and an account of all patients [24]. The maximum points of each parts are three, three, and one, respectively. Publications that got three points total are high-quality studies [24].

Finally, as for the 4821 utility measurements from the 122 selected longitudinal studies, the random effect models with robust estimation [25] were applied to synthesize the impact of these interventions on the QoL point of cancer patients when conducting the meta-regression. We did not use restricted maximum or maximum likelihood because we collected all data in different research. However, each research they used dissimilar methods and various scale to assess the quality of life points. Therefore we can only group these scales in different groups and applied the random effect models analysis.

There are several research-related factor groups that need to be analysed, so when we consider using the funnel plot and forest plot, it may infeasible. Moreover, the amount of collected data was numerous, then we believed that the random effect models with meta regression can be used to evaluate these factors systematically. Specifically, these models can synthesize the relationship between several feature groups such as types of cancer, types of intervention, and types of country, health utility measurements, and study design to assess the changes in health utility points over time. The meta regression analysis by using STATA software version 16.0 (StataCorp LLC, College Station, TX) was applied to evaluate the change in the health utility scores in each model. Elements are divided into different models: measure, types of cancer, types of interventions, country types and study designs. Within each group, we compared factors with each other and assessed the change in different models. In particular models, the change between quality of life scales is evaluated to find statistical significance. The P-value in this meta regression analysis was designed at the threshold of 0.01, 0.05, and 0.1, respectively, as three levels of evaluation.

## Results

### The quality of included longitudinal studies

#### The Newcastle –Ottawa Scale

Table 2 shows the data on the quality of research studies in all the analysed topics except for Randomised control trials. These studies were analysed using the Newcastle-Ottawa Scale. In general, the studies are assessed into three groups namely Good, Fair, and Poor. The studies have fair quality, and they were the highest figure, with 44 studies. This group took up 56.41% of the total research studies with a mean point of 6.09 and a standard deviation (SD) of 0.60. The number of good-quality studies is ranked second on the list with 31 studies accounting for nearly 40%. The mean point of this group is 7.71 and the SD is 0.44. The final group of studies has poor quality, and only accounts for 3.85% of the studies analysed. The mean point and SD of this group were 5.00 and 1.00, respectively.

**Table 2:** The Newcastle-Ottawa Scale point of studies included

Quality	n	%	Mean point	SD
Good	31	39.74	7.71	0.84
Fair	44	56.41	6.09	0.60
Poor	3	3.85	5.00	1.00

#### The Jadad scale

Table 3 depicts the data of studies conducted based on the design of randomized control trials. These data were obtained using the Jadad Scale to analyse the quality of the studies. According to the results of this scale, the studies were divided into two groups namely High and Low quality, with over two third of them being High-quality. There were 30 High-quality studies, taking up 68.18% of the total number of studies on the topic of randomized control trials. The mean point of this group was 3.43 with 0.77 points of SD. For the Low-quality studies, 31.82% were classified into this group with 14 studies. The mean point of this group was lower than the former group at only 1.57 with an SD of 0.67.

**Table 3:** The Jadad Scale point of studies included

Quality	N	%	Mean point	SD
High	30	68.18	3.43	0.77
Low	14	31.82	1.57	0.67

### The characteristics of selected utility measurements

Table 4 describes the characteristics and usage frequency of different health utility measurements in the 122 selected studies. Overall, there are six main features of the methods used by the articles considered in this meta-analysis including Time of administration, Measures, Country, Study design, Intervention, and Type of cancer. The data revealed that nearly half (46.32% − 2233 times) of the health utility measurements were collected over 6–12 months. Measurements made within 6 months only account for nearly half as much (22.36% − 1078 times). For more than 24 months and 12–24 months, there were 884 and 626 times in which the measurements were used in research conducted, accounting for 18.34% and 12.98% of total measurements, respectively.

**Table 4:** The characteristic of health utility measurements

Category	Characteristic	Number of utility measures (*n* = 4821)	
		n	%
**Time of administer**	< 6 months	1078	22.36
	6–12 months	2233	46.32
	> 12–24 months	626	12.98
	> 24 months	884	18.34
**Measures**	EORTC	3141	65.15
	FACT	584	12.11
	SF-36	466	9.67
	QLI	59	1.22
	FLIC	55	1.14
	EQ-5D	37	0.77
	Others	479	9.94
**Country**	Sweden	929	19.27
	USA	621	12.88
	China	477	9.89
	Netherland	362	7.51
	Germany	358	7.43
	Canada	258	5.35
	Norway	227	4.71
	France	148	3.07
	Denmark	142	2.95
	The UK	124	1.16
	Others	609	25.78
**Study design**	Longitudinal	2468	51.19
	Cohort	687	14.25
	Case-control	256	5.31
	Randomized control trial	1410	29.25
**Intervention**	Pharmacological group	3019	62.62
	Non-pharmaceutical group	1802	37.38
**Type of cancer**	Breast Cancer	787	16.32
	Lung Cancer	737	15.29
	Head and Neck Cancer	688	14.27
	Research evaluating more than one type of cancer	540	11.20
	Prostate Cancer	406	8.42
	Esophageal or gastroesophageal junction	230	4.77
	Ovarian Cancer	135	2.80
	Gastric Cancer	122	2.53
	Others	1176	24.39

Regarding the measurement metrics, there were several scales used to quantify the quality of life of cancer patients in the analysed research. In this table, seven main scales have been listed. EORTC ranked first on the list with a vast majority of 3141 times mentioned, taking up 65.15%. This figure was nearly sixfold that of the next most used metric, FACT, which accounted for 584 times and 12.11% of total measurements. SF-36 was the third most popular metric, taking up 9.67% of total measurements with 466 times used. The scales QLI, FLIC, EQ-5D, and VAS took up a very small portion, at 1.22%, 1.14%, 0.77%, and 0.48%, respectively. Other scales that were used in studies cumulatively accounted for 9.46% of total measurements.

The third feature of the analysed papers was the country in which the research was conducted. Top of the list was Sweden: research conducted in Sweden collected 929 health utility measurements which took up 19.27%. The USA ranked second, accounting for 12.88% with 621 measurements. China was the only Asian country on the list which ranked third place with 477 measurements taking up 9.89%. Ranked from fourth to tenth place on the list were six other countries namely Netherlands, Germany, Norway, France, Denmark, and the UK. Canada ranked sixth on the list with 258 measurements.

There were four types of study design Longitudinal, Cohort, Case-control, and Randomized control trials. Accounting for over haft of the total usage of health utility measurements was Longitudinal studies with 2468 measurements The second most used study design was a Randomized control trial with 1410 measurements. The third place was the cohort study, which collected 687 measurements, accounting for 14.25%. Finally, the case-control study design took up only 5.31% with 256 measurements.

The use of health utility measurements in Pharmacological interventions was far more popular than non-pharmaceutical ones. To be more specific, 62.62% of the health utility measurements in published papers on the topic of QoL of cancer patients were for pharmacological interventions (3019 measurements) compared to 37.38% (1802 measurements) from non-pharmaceutical interventions.

Finally, there were eight main kinds of cancer investigated in the selected studies. Cancer with the most measurements is breast, lung, and head and neck cancer with 787 (16.32%), 737 (15.29%), and 688 measurements respectively. Next, research evaluating more than one type of cancer and Prostate cancer was measured 540 and 406 times, respectively. Other types of cancer were Esophageal or gastroesophageal junction, Ovarian Cancer, and Gastric Cancer. The total proportion of these three types of cancer was 10.1%. Other types of cancer were cumulatively categorized as Others and took up 24.39% of the total number of times health utility measurements with 1176 times.

### Adjusted effect size models

In Table 5, we evaluated the change in the quality-of-life point through different models with the following factors: types of cancer, types of interventions, country type, and study design.

**Table 5:** The adjusted effect models

	n	%	Model 1 Health utility scale	Model 2 Types of cancer	Model 3 Types of interventions	Model 4 Country types	Model 5 Study designs
			Coeff. (95% CI)	Coeff. (95% CI)	Coeff. (95% CI)	Coeff. (95% CI)	Coeff. (95% CI)
Const			47.519***	43.481***	34.610***	47.339***	47.447***
			(21.051–73.987)	(20.399–66.563)	(10.496–58.724)	(20.998–73.681)	(20.861–74.033)
Measure							
EORTC	3141	65.15	0.184	-1.610	4.285	0.040	-0.921
			(-26.512–26.881)	(-26.576–23.356)	(-19.078–27.648)	(-26.512–26.591)	(-27.934–26.092)
FACT	584	12.11	-2.111	-6.564	1.557	-2.293	-3.578
			(-31.500–27.279)	(-32.908–19.779)	(-23.929–27.042)	(-31.891–27.305)	(-33.276–26.119)
SF36	466	9.67	27.583	16.859	30.574*	26.836	24.989
			(-8.753–63.918)	(-14.484–48.203)	(-0.489–61.636)	(-9.600–63.271)	(-12.163–62.141)
QLI	59	1.22	-4.078	-2.128	-2.855	-4.236	-5.215
			(-34.823–26.667)	(-33.027–28.772)	(-30.749–25.040)	(-34.783–26.311)	(-35.977–25.548)
FLIC	55	1.14	31.146	23.858	41.435*	30.254	29.797
			(-20.313–82.605)	(-26.729–74.445)	(-6.886–89.757)	(-20.421–80.928)	(-20.825–80.419)
VAS	23	0.48	-43.518**	-46.011**	-41.824**	-43.561**	-43.851**
			(-79.783 - -7.252)	(-83.782 - -8.239)	(-76.787 - -6.862)	(-79.765 - -7.358)	(-80.270 - -7.432)
Other	456	9.46	-24.614*	-29.579**	-21.882*	-25.147*	-25.409*
			(-50.459–1.231)	(-52.579 - -6.580)	(-44.499–0.735)	(-50.800–0.506)	(-51.439–0.620)
Types of cancer							
Breast cancer	787	16.32		6.310			
				(-7.831–20.451)			
Head and Neck cancer	688	14.27		-0.615			
				(-13.797–12.567)			
Research evaluating more than one type of cancer	540	11.20		5.158			
				(-6.084–16.400)			
Prostate cancer	406	8.42		26.599***			
				(12.554–40.643)			
Esophageal or gastroesophageal junction cancer	230	4.77		16.410***			
				(5.891–26.928)			
Ovarian cancer	135	2.80		10.208			
				(-18.694–39.109)			
Gastric cancer	122	2.53		27.848*			
				(-3.809–59.505)			
Gastrointestinal cancer	119	2.47		-4.927			
				(-13.258–3.404)			
Endometrial Cancer	114	2.36		-5.708			
				(-18.357–6.942)			
Nasopharyngeal carcinoma	90	1.87		4.845			
				(-5.746–15.436)			
Esophageal cancer	84	1.74		16.134			
				(-18.555–50.822)			
Cervical cancer	80	1.66		-18.021***			
				(-28.612 - -7.430)			
Leukemia	77	1.60		26.837*			
				(-3.725–57.398)			
Gynecological Cancer	73	1.51		14.543			
				(-8.913–37.998)			
Colorectal cancer	71	1.47		20.009***			
				(9.418–30.600)			
Pancreatic cancer	60	1.24		-0.251			
				(-10.842–10.340)			
laryngeal cancer	58	1.20		5.969			
				(-10.065–22.003)			
Sarcoma	57	1.18		-6.667			
				(-17.258–3.924)			
Malignant Melanoma	54	1.12		-17.089			
				(-49.960–15.782)			
Rectal cancer	53	1.10		-8.052			
				(-18.643–2.539)			
Hodgkin Lymphoma	42	0.87		29.844***			
				(19.253–40.435)			
Eye cancer	30	0.62		6.765			
				(-3.826–17.356)			
Rectal cell cancer	30	0.62		-0.208			
				(-10.799–10.383)			
Carcinoma	28	0.58		-4.324			
				(-14.915–6.267)			
Brain cancer	18	0.37		41.822***			
				(33.388–50.257)			
Bone cancer	6	0.12		6.672			
				(-13.840–27.184)			
Acute myeloid leukemia	2	0.04		95.083***			
				(81.332–108.835)			
Kidney cancer	2	0.04		-43.241***			
				(-66.323 - -20.159)			
Types of interventions							
Chemotherapy + Radiotherapy	623	12.92			0.703		
					(-13.855–15.260)		
Psychological support program	611	12.67			4.729		
					(-8.024–17.482)		
Radiotherapy	368	7.63			30.588***		
					(17.327–43.850)		
Palliative care	336	6.97			18.355**		
					(3.308–33.403)		
Chemotherapy + Radiotherapy + Surgery	255	5.29			-0.395		
					(-11.495–10.705)		
Radiotherapy + surgery	250	5.19			-6.789		
					(-26.329–12.750)		
Physical exercise	236	4.90			13.728**		
					(0.180–27.275)		
Combine curative and palliative care	218	4.52			23.787**		
					(2.813–44.762)		
Chemotherapy + surgery	182	3.78			4.846		
					(-4.855–14.548)		
Psychological group therapy	165	3.42			-3.831		
					(-12.328–4.666)		
Short term psycho-educational	110	2.28			-0.648		
					(-9.118–7.823)		
Stem Cell Transplantation	80	1.66			4.324		
					(-15.740–24.388)		
Complementary Alternative therapy	66	1.37			5.050		
					(-5.189–15.290)		
Best supportive care + chemotherapy	60	1.24			59.782***		
					(48.743–70.820)		
Screening	51	1.06			31.006***		
					(18.284–43.729)		
Anti-hormone Therapy	48	1.00			-2.414		
					(-17.791–12.962)		
Country type							
Upper middle-income country	484	10.04				3.645	
						(-9.238–16.528)	
Study design							
Randomized control trials	1410	29.25					2.321
							(-7.952–12.593)
Cohort study	687	14.25					4.912
							(-4.602–14.426)
Case-control study	256	5.31					9.143
							(-19.333–37.619)

Robust ci in parentheses

*** *p* < 0.01, ** *p* < 0.05, * *p* < 0.1

In model 1, when comparing the quality of life assessment scales with the first scale, EQ-5D, the results showed that the VAS scale (*p* < 0.05) had significant statistics. We chose the EQ-5D scale as the control because it was one of the most basic and useful scales in assessing the health utility of patients for more than 30 years. Besides, it can assess the quality of life of patients with both physical and mental characteristics [26].

With model 2, we compared health utility measurements with types of cancer. The results showed that there are statistically significant changes in several cancer groups such as acute myeloid leukemia, brain cancer, cervical cancer, colorectal cancer, oesophageal or gastroesophageal junction cancer, Hodgkin lymphoma, kidney cancer, prostate cancer (*p* < 0.01) and leukemia, gastric cancer (*p* < 0.1). This model also showed that the VAS scale and other scales performed significantly differently from EQ-5D (*p* < 0.05). Model 3 compared different intervention groups and radiotherapy, screening, combine supportive care and chemotherapy were found to have a statistically significant change (*p* < 0.01). Besides, the palliative care group, the combined curative and palliative care group, and the physical exercise group were also statistically significant with *p* < 0.05. Additionally, model 3 witnessed a significant change in measurement scale with SF36, FLIC, and others (*p* < 0.1), especially with the VAS scale (*p* < 0.05). When comparing the results of the upper middle-income countries and high-income countries, we did not see a statistically significant difference between them. Finally, there were also no statistically significant differences between the study design groups.

## Discussion

### The quality of full-text paper

In this study, two types of paper quality assessment scales were applied, and we found that 122 included papers have good and fair quality in general. As regards The Newcastle-Ottawa Scale is useful to evaluate longitudinal studies with non-randomized control trial design. With this scale, we found that nearly 40% of included studies were good while 60% were fair. The primary reasons are the lack of information on the selection, exposure, and outcomes sections. Some studies did not have specific criteria for selecting subjects, or reasons for selecting subjects in the selection. In addition, studies did not provide detailed information on how outcomes and exposure were collected and evaluated at the end of the process. The results of our study are similar to the systematic review of chemotherapy and surgery’s impact on the quality of life of breast cancer patients in 2022. Among 26 studies collected, 34% were good and 66% were fair [27]. However, another systematic review and meta-analysis in 2020 researched on survival rate in colorectal cancer and they found that 60% of the collected paper was at a good rate and 40% at a fair rate [28]. Although the majority of included studies getting medium or high quality, there are still some studies that need to be considered more carefully when developing a research design, analysing the data, and presenting the results.

The other type of quality assessment scale, the Jadad Scale, determined that approximately 70% of randomized control trial studies had high quality and only one-third of them had low quality. When we scored the included studies, we found that some of the articles lack information in the description of randomized and blinding criteria. They did not specify the reasons for selection or the method of selecting research subjects into random groups or did not specify the method for blinding criteria. Some articles did not mention whether they blinded the subject. The results of our study are higher than a systematic review in 2013 that researched the effectiveness of palliative care in cancer patients. The reason for this difference may be that the above author’s study only focused on evaluating randomized control trials (RCTs) conducted in China without other countries [29]. Another systematic review conducted in 2017 also used Jada’s scale to evaluate the quality of RCTs and found that there were 10/18 (55.6%) studies with Good quality which is lower than our study [30]. However, the authors only focused on evaluating studies that applied an intervention or pain management and were conducted in Africa, Europe, and North America.

Although the systematic review and meta-analysis mentioned above are conducted in different sets of studies, the quality evaluation of included studies may be essential. It might give information about the current trends in research design and present, especially with the rapidly increasing amount of research being published. We believed that it is essential to apply scales such as the NewCastle-Otawa and Jadad Scale to evaluate them carefully.

### The characteristic of included longitudinal research

Of the 122 studies selected for meta-regression analysis, we found that nearly 50% of the studies were designed with a 6–12 month follow-up period. At this point, if the follow-up time is too short, it may not fully reflect the change in the patient’s QoL indicators. However, if the follow-up time is too long, it can lead to the loss of patients because of cancer and especially metastatic cancer, the patient’s survival rate will change from time to time. The loss of patients can lead to biases in the evaluation of longitudinal research. However, in a long time of follow-up, they may have a better assessment of the change in the health utility of cancer patients because they will not miss the decrease in the quality of life of cancer patients in their last days. Consequently, these follow-up assessments are often difficult to achieve high accuracy avoiding biases.

In this research, we counted about three hundred single tools used to evaluate different aspects of the health utility of cancer patients. These tools can be classified into eight main groups named: EQ-5D, VAS, EORTC-QLQ, FACT, FLIC, SF36, QLI, and others. The most used scale was EORTC-QLQ. However, the EORTC scale can be designed with many other subgroups such as EORTC QLQ C36, EORTC QLQ HN37, EORTC QLQ HN35, EORTC QLQ OV28, EORTC QLQ BR23, EORTC QLQ BR38, and EORTC QLQ LC13 which are used to assess different types of cancer. We found that another group of authors who conducted a systematic review of 13 articles on the health-related QoL of prostate cancer patients claimed that 7/13 articles used the EORTC scales [31]. In addition, another research in 2021 also assessed the impact of physical therapy on oesophageal cancer patients with all the included studies using the EORTC scale [32]. This result is different from our study but is due to the huge difference in the number of studies evaluated. Although the EORTC-QLQ scale is very useful, it is necessary to compare different scales, especially with some other important scales such as EQ-5D, VAS, FACT, and SF- 36.

On the other hand, FACT is another important scale with nearly six hundred measurements made with FACT in the 122 included studies. This scale also has several subgroups and tools, which can be modified for different types of cancer intervention impact assessment. In another study, besides the EORTC scale, FACT is also commonly used with a ratio of 26/43 of the researches [16]. Therefore, FACT is one of the most common scales which may be needed more in-depth research.

Regarding the contribution of different countries in this research field, Sweden ranked at top of the list with nearly a thousand of these tools. Moreover, the data showed that America and China still had a high number of QoL measurements, followed by other Western European countries and Canada. It is difficult to compare our results with previous publications because our research was not limited to any region or country in the world.

In addition, there was a significant difference between the pharmacological and non-pharmaceutical interventions. The non-pharmaceutical intervention has only been the focus of research in recent years. However, this intervention group still played an important role in the improvement of the well-being of cancer patients. As for the type of cancer, lung cancer was the highest rank with more than seven hundred health utility measurements. It was followed by breast cancer, head, and neck cancer, prostate cancer, and other types. These were all cancers with high incidence rates with several burdens on the QoL of cancer patients. Therefore, the majority of research conducted so far often focused on these diseases. In a systematic review of the impact of exercise on patients with various cancers conducted in 2017, the most frequent cancers, in descending order, were found to be: breast cancer, gastrointestinal, head and neck cancer, cancer, endometrial and ovarian cancer, prostate cancer, lung cancer, blood cancer, and others. There is a remarkable difference in the rank of cancer types compared to our study, but this difference is due to our comprehensive approach to types of cancer intervention [33]. In this systematic review, we realized that some cancers such as breast cancer, lung cancer, and prostate cancer had a higher research focus. Whereas some other types of cancer are uncommon, the sample size is small, and the research and evaluation can appear less often. This is similar to the group of interventions, the studies mainly only evaluated some common methods such as screening, chemotherapy, radiotherapy, surgery, palliative care, and a combination of them. Thus, more future studies are needed to evaluate more about some rare cancer types and new modern interventions.

### The adjusted effect models

Our group is one of the first to apply random effect models to investigate the change in the health utility point of cancer patients. We evaluated and compared the impact of the group of quality of life measures, and the group of measures against other factors such as types of cancer, types of intervention, country types, and study design. The results showed that there are statistically significant changes when comparing groups of health utility measurement tools, cancer types, and interventions. Among them, we found some prominent scales such as VAS. This scale exhibited some differences when included in the comparison models. In a systematic review of self-report instruments for the measurement of anxiety in hospitalized children with cancer in 2021, the authors also recommend that the VAS scale be combined with other self-report scales [34]. Besides, commonly used scales including EORTC and FACT have no statistically significant difference when included in the models compared to the scale EQ-5D. However, the role of these scales in cancer intervention research is essential. In the systematic review in 2011, the authors suggested that the EORTC and FACT scale rank at the top of the list for impact scales [35]. This research suggested that further evaluation of the VAS scale will need to be done to determine its effectiveness in scoring patients’ QoL.

Besides, several cancer groups including acute myeloid leukemia, brain cancer, colorectal cancer, oesophageal or gastroesophageal junction cancer, Hodgkin lymphoma, and prostate cancer had a positive statistically significant change. On the other hand, cervical cancer and kidney cancer had negative statistically significant changes in model of type of cancer. Cancer patients in these groups are often monitored for changes in the QoL point continuously over a certain period along with the impact of interventions. From there, scientists can further evaluate changes in QoL points with different influencing factors.

In addition, when evaluating the interventions, we found that the radiotherapy group, screening, combined supportive care and chemotherapy, palliative care group, combined curative and palliative care group and physical exercise group elicited significant changes in the quality of life of patients. This result emphasizes the need for close coordination between pharmacological and non-pharmaceutical interventions to improve the quality of life for cancer patients. Curative care interventions still play an important role to help the patient’s QoL, especially radiotherapy. However, the results also indicated that major changes were often concentrated in groups that combine multiple approaches between pharmaceutical and non-pharmacological interventions. Indeed, best supportive care combined with chemotherapy and curative combined with palliative care groups showed statistically significant changes in the QoL score when apply to cancer patients during the time of follow-up.

A systematic review in 2017 indicated that patients with comorbidities and chemotherapy had decreased HRQOL after treatment [16], however, the authors suggested that the combination of additional palliative care and social support can improve the quality of life of cancer patients. While we found significant differences in QoL scores when comparing cancer types, interventions, and influencing factors such as country types and study design when applying the different scales. Nonetheless, there is no significant statistic when analysing by country type and study designs. Thus, these factors may not be the primary factors affecting changes in health utility scores.

However, there were some limitations in our research, including the stage of cancer, the demographic of participants, and several types of high-technology intervention that were not analysed in our results such as targeted drugs, new gene sequencing, and immunotherapy. Thus, future researchers should consider these aspects and conduct more research with different types of biostatistical analysis models. The above factors can play an important role in the change of the health utility point of cancer patients under different interventions. However, the random effect models were chosen because we believe that it may be effective on analysing the huge amount of data and the variety of vital affecting factors. In addition, the sensitivity was not analysed as one of the limitation of us. However, it should be considered carefully in the further research.

## Conclusion

Based on these results, most papers have good or fair quality, however, some of the included studies may have to be developed carefully. Besides, the EORTC QLQ and FACT scale should be focused on in future research. The results also suggested that some common and dangerous types of cancer are being focused on by several developed countries. Then, it can be an opportunity for future researchers to analyze deeper the features of the country and types of cancer. Additionally, the adjusted effect model described the change of health utility scores in different models that compare the effect of the associated factors and found that the VAS scale had significant statistics in all models when compared with the EQ-5D scale. Consequently, further specific research should be done to compare these scales with different aspects. Our research also found some differences in the types of cancer, health utility measurements, and types of interventions, but not found in country types and study design. Despite some limitations, we believe that this research can provide general and basic information about the impact of cancer interventions to improve the quality of life of cancer patients in some previous longitudinal research. Therefore, it can suggest broadening the research area with some vital features of cancer, populations, and high technology interventions in the future.

### Supplementary Information

**Additional file 1: Appendix 1.** Searching keyword terms

## Data Availability

The datasets used and/or analyzed during the current study are available from the corresponding author on reasonable request.

## Abbreviations

QoL: Quality of life
HRQL/ HRQoL: Health-related quality of life
WHO: World Health Organization
PRISMA-P: Preferred Reporting Items for Systematic Review and Meta-Analysis Protocol
WOS: Web of science
