# Supplementary material for: Impact of interventions on the quality of life of cancer patients: a systematic review and meta-analysis of longitudinal research
Source: Health Qual Life Outcomes. 2023 Oct 11;21:112. doi: 10.1186/s12955-023-02189-9 (PMC10566122; doi:10.1186/s12955-023-02189-9)
Supplement: Supplementary file 1 — Additional file 1: Appendix 1. Searching keyword terms [file 12955_2023_2189_MOESM1_ESM.docx]

**Appendix 1: Searching keyword terms**

**Table 1: The keyword terms for searching**

| **Terms** | **Keywords** |
| --- | --- |
| Term #1 | **Quality of life**: TS = “quality of life” OR TS = “well-being” |
| Term #2 | **Cancer**: TS= “cancer*” OR TS = “metasta*” OR TS = “Oncolog*” OR TS = “oncogen*” OR TS = “carcinogen*” OR TS = “maglinan*” OR TS = “Tumor*” OR TS = “Tumour*” OR TS = “Astrocytoma” OR TS = “Atypical Teratoid” OR TS = “Blastoma” OR TS = “Carcino*” OR TS = “Cholangiocarcinoma” OR TS = “Chordoma” OR TS = “Craniopharyngioma” OR TS = “Ependymoma” OR TS = “Erythroplasia” OR TS = “Esthesioneuroblastoma” OR TS = “Gestational Trophoblastic Disease” OR TS = “Histiocyto*” OR TS = “Leukemia” OR TS = “Lymphoma” OR TS = “Melanoma*” OR TS = “Mesothelioma” OR TS = “Myelo*” OR TS = “Neoplas*” OR TS = “Neuroblastoma” OR TS = “Neurofibromato*” OR TS = “Osteosarcoma” OR TS = “Paraneoplastic” OR TS = “Pheochromocytoma” OR TS = “Rhabdoid” OR TS = “Retinoblastoma” |
| Term #3 | **Intervention**: TS= “intervention” OR TS= “trial” OR TS= “interventions” OR TS= “trials” |
| Term #4 | #1 AND #2 AND #3 |
